# Regularized Denoising Masked Visual Pretraining for Robust Embodied PointGoal Navigation

**DOI:** 10.3390/s23073553

**Published:** 2023-03-28

**Authors:** Jie Peng, Yangbin Xu, Luqing Luo, Haiyang Liu, Kaiqiang Lu, Jian Liu

**Affiliations:** 1 Institute of Microelectronics of the Chinese Academy of Sciences, Beijing 100029, China; pengjie@ime.ac.cn (J.P.); xuyangbin@ime.ac.cn (Y.X.); luoluqing@ime.ac.cn (L.L.); liuhaiyang@ime.ac.cn (H.L.); lukaiqiang@ime.ac.cn (K.L.); 2 University of Chinese Academy of Sciences, Beijing 100049, China

**Keywords:** robust visual navigation, Kullback–Leibler divergence, denoising, masked visual pretraining, self-supervised learning, vision transformer, embodied AI

## Abstract

Embodied PointGoal navigation is a fundamental task for embodied agents. Recent works have shown that the performance of the embodied navigation agent degrades significantly in the presence of visual corruption, including Spatter, Speckle Noise, and Defocus Blur, showing the weak robustness of the agent. To improve the robustness of embodied navigation agents to various visual corruptions, we propose a navigation framework called Regularized Denoising Masked AutoEncoders Navigation (RDMAE-Nav). In a nutshell, RDMAE-Nav mainly consists of two modules: a visual module and a policy module. In the visual module, a self-supervised pretraining method, dubbed Regularized Denoising Masked AutoEncoders (RDMAE), is designed to enable the Vision Transformers (ViT)-based visual encoder to learn robust representations. The bidirectional Kullback–Leibler divergence is introduced in RDMAE as the regularization term for a denoising masked modeling task. Specifically, RDMAE mitigates the gap between clean and noisy image representations by minimizing the bidirectional Kullback–Leibler divergence. Then, the visual encoder is pretrained by RDMAE. In contrast to existing works, RDMAE-Nav applies denoising masked visual pretraining for PointGoal navigation to improve robustness to various visual corruptions. Finally, the pretrained visual encoder with frozen weights is applied to extract robust visual representations for policy learning in the RDMAE-Nav. Extensive experiments show that RDMAE-Nav performs competitively compared with state of the arts (SOTAs) on various visual corruptions. In detail, RDMAE-Nav performs the absolute improvement: 28.2% in SR and 23.68% in SPL under Spatter; 2.28% in SR and 6.41% in SPL under Speckle Noise; and 9.46% in SR and 9.55% in SPL under Defocus Blur.

## 1. Introduction

In recent years, Embodied AI [1] has received extensive attention; it requires the agent to complete a specific task by interacting with the environment. As one of the specific tasks, PointGoal navigation has made great progress with the development of deep reinforcement learning (DRL), computer vision and robotics, and the emergence of a large number of photo-realistic simulation platforms [2,3,4]. PointGoal navigation is one of the most fundamental and important tasks in Embodied AI, and it is also the basis for the embodied agent to complete other more difficult tasks. In PointGoal navigation, an agent is required to move from its current position to a given coordinate point in 3D environments by using egocentric RGB (or RGB-D) observations and GPS+Compass localization only [5].

Traditional navigation methods, such as SLAM (Simultaneous Localization and Mapping)-based navigation methods, rely on environmental prior maps and high-precision sensors. Therefore, they exhibit poor generalization for prior unknown environments [6,7]. These difficulties have motivated a flux of research into DRL-based visual navigation techniques, which provide an end-to-end map-free navigation approach. By leveraging the powerful visual representation and decision-making capabilities of DRL, visual navigation is able to understand the environment with inexpensive sensors (such as monocular RGB cameras) and make full use of previously seen environments to learn knowledge for unseen environments’ generalization.

So far, a lot of work has been performed for PointGoal visual navigation [8,9,10,11,12]. Most of these efforts focus on improving generalization to novel environments, where the agent’s egocentric RGB observations are without visual corruption on evaluation. However, the ultimate purpose of the PointGoal navigation agent is to work in real environments with large variations in visual corruptions, e.g., the cameras are occluded by water droplets.

**Figure 1:**
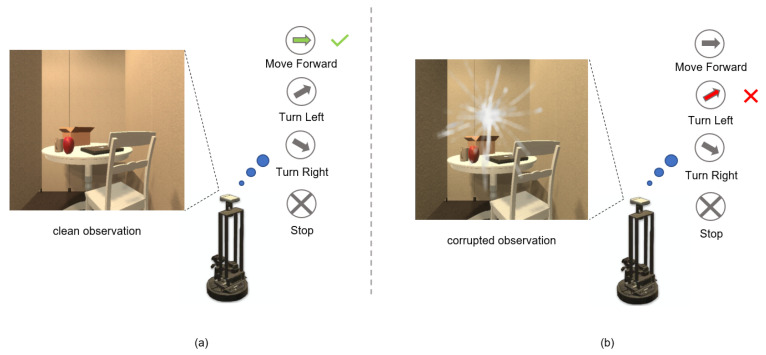
Illustration of navigation under clean/corrupted observation. (**a**) Optimal action decision under clean observation. (**b**) Non-optimal action decision under corrupted observation.

To evaluate the robustness of embodied navigation agents in various visual corruptions, ROBUSTNAV [13] provides a variety of realistic visual corruptions for emulating corruptions in the real world, and the robustness of the agent is evaluated after learning navigation policies with those visual corruptions. Ref. [13] points out that the performance of standard navigation agents, which work effectively with clean observation, drops dramatically in the presence of visual corruption. As shown in Figure 1a, the navigation agent’s observation is clean, and it is straightforward to choose the optimal action. However, it is more difficult for the agent to choose the optimal action in the presence of visual corruption, as shown in Figure 1b. It is worth noting that the agent has to choose an action at each time step in navigation. The weak robustness to visual corruption causes the agent to struggle to reach the target.

To address this issue, we propose a novel Regularized Denoising Masked AutoEncoders Navigation framework (RDMAE-Nav), which is a robust PointGoal navigation agent for various visual corruptions. To be specific, RDMAE-Nav mainly consists of two modules: a visual module and a policy module. In the visual module, a Vision Transformer (ViT)-based [14] visual encoder is introduced to extract features of the agent’s egocentric RGB observations. Moreover, a novel pretraining method is designed to learn robust visual representations, which is called Regularized Denoising Masked AutoEncoders (RDMAE). Inspired by the denoising mechanism in DMAE [15], which shows great robustness in image classification, the proposed RDMAE feeds both masked clean and noisy images in the encoder–decoder scheme to reconstruct the original clean images in a self-supervised way, where the two latent feature representations from clean and noisy images are mitigated via a constructed regularized loss by evaluating the bidirectional Kullback–Leibler (KL) divergence. Then, the visual encoder is pretrained to obtain robust and efficient visual priors via RDMAE, which provides a denoising masked modeling task with a regularized term. After the pretraining, the encoder of the RDMAE is taken as the visual encoder of the proposed RDMAE-Nav. Noteworthily, in the subsequent navigation policy learning of RDMAE-Nav, the pretrained visual encoder is applied to extract robust representations of the agent’s RGB observations only, and its parameters are not updated. To the best of our knowledge, RDMAE-Nav is the first attempt to exploit denoising masked visual pretraining for PointGoal navigation to improve robustness to various visual corruptions. As a result, the visual module encodes the agent’s egocentric RGB observations into the visual embedding. In the policy module, the goal localization is encoded into the goal embedding. Accordingly, the visual embedding, the goal embedding, and the previous hidden states are aggregated into the navigation policy network. The policy network takes advantage of the memory-capable Gated Recurrent Unit (GRU) [16] network to make future decisions based on past information. Additonally, the Decentralized Distributed Proximal Policy Optimization (DD-PPO) [8] paradigm is adopted to learn the policy network.

By following ROBUSTNAV [13], the experiments are conducted on the AI2THOR simulation platform [2], which provides a vast navigation environment with large variations. The results of robust navigation evaluation show the efficacy of our contribution and the advantages of our method over existing methods in a variety of visual corruptions. Overall, our contributions are summarized as follows:

- We propose a robust PointGoal navigation framework RDMAE-Nav as the first attempt to apply denoising masked visual pretraining for embodied PointGoal navigation, which is a robust navigation agent for various visual corruptions.
- We design a novel pretraining method, dubbed RDMAE, which introduces a regularization term for a denoising masked modeling task. RDMAE mitigates the gap of representation distributions between clean images and noisy ones by minimizing the bidirectional Kullback–Leibler (KL) divergence and consequently enables the visual encoder to obtain more robust and efficient visual priors.
- Our method can achieve competitive performance over all competitors through experiments on the ROBUSTNAV benchmark [13], demonstrating the effectiveness and efficiency of the proposed RDMAE-Nav by employing Regularized Denoising masked visual pretraining for various visual corruptions.

## 2. Related Work

### 2.1. Embodied PointGoal Navigation

With the emergence of a large number of simulation platforms, such as Habitat [17] and AI2THOR [2], PointGoal navigation tasks have a standard dataset, agent configuration, and evaluation metrics, which have greatly boosted the research progress of PointGoal navigation. A landmark work is [8], whose agent architecture consists mainly of a visual encoder and a policy network. The visual encoder uses ResNet50 to extract features of the RGB-D visual input. The policy network consists of a two-layer Long Short-Term Memory (LSTM) [18] that takes the previous action, the localization information, and the output of the visual encoder as input. By proposing the DD-PPO algorithm, the training process of distributed reinforcement learning in a resource-intensive simulation environment is greatly accelerated. After about 2.5 billion frames of training, it achieved SOTA results at the Habitat Autonomous Navigation Challenge 2019, and the results are near-perfect. However, this approach relies on a large amount of computation and requires about 6 months of GPU time for training, which is unaffordable for the average researcher.

To improve sample efficiency and save computational resources, [9] investigated the PointGoal navigation method under resource constraints (specifically about 75 million frames and 1 GPU for 1 day). It adopts the more lightweight ResNet18 as the visual encoder and proposes not to use Generalized Advantage Estimation (GAE) [19] in the training and to use a larger batch size. Experimental results indicate that the performance of this approach can even outperform the method proposed by [8] in the resource-constrained condition.

An important reason why PointGoal navigation achieves near-perfect performance is that the simulation environment has perfect localization, which is usually difficult to satisfy in the real environment. Ref. [10] used visual odometry to replace the GPS+Compass sensor and achieved surprising performance on the Habitat PointNav benchmark. Ref. [11] combined classical SLAM approaches with learning-based approaches and proposed a learning-based differentiable SLAM approach to achieve State-Of-The-Art (SOTA) performance in the Habitat 2020 PointNav challenge. Ref. [12] proposed the use of a self-supervised method for monocular depth estimation to replace depth sensors on the PointNav navigation task and achieved excellent performance. There are also some works [20,21] studying sim2real, where they train the agent in a simulation environment and then use it in a real environment.

Most of the current work focuses on improving the generalization of the agent to novel environments, and the agent’s visual observations are clean during training and validation. However, there are various visual corruptions in real environments. Although [10,22,23] considered the visual sensors and actuation noise that may exist in navigation, these noise types are too simple to cover the rich visual corruptions that exist in real environments. In order to be able to quantitatively evaluate the robustness of embodied navigation agents to visual corruptions that may exist in the real environment, [13] proposed an evaluation framework called ROBUSTNAV. ROBUSTNAV contains a variety of visual corruptions, including Spatter, Speckle Noise, Camera Crack, Lower FOV, Defocus Blur, Motion Blur, etc. These visual corruptions can simulate real-world perturbation scenarios well. Ref. [13] pointed out that the performance of some near-perfect methods is severely degraded when evaluated in the presence of visual corruption.

### 2.2. Pretrained Visual Encoders in Embodied Visual Navigation

In recent years, pretrained visual encoders have been widely used in visual navigation. Compared with training from scratch, pretrained visual encoders that introduce visual priors lead to higher sample efficiency and better generalization of the navigation agent [24]. In [24], using midlevel visual representations to learn navigation policies instead of learning directly from the raw agent’s visual observations is proposed. Specifically, it pretrained the visual encoder on specific visual tasks and then used its freeze weights to extract visual representations from the raw images. Extensive experiments have demonstrated that using a pretrained visual encoder can greatly improve the sample efficiency and generalization of the navigation agent. In [25], the visual encoder of the navigation agent was allowed to be pretrained on a self-supervised environment prediction task. The pretrained visual encoder was able to learn the spatial representation of the environment and could be better used for downstream navigation tasks. In [26], VTNet (Visual Transformer NetWork) was used to correlate visual representations with navigation signals for visual pretraining, which accelerated the learning on navigation policy. In [27], a CLIP (Contrastive language image pretraining) [28] encoder with frozen weights was directly used as a visual encoder without any fine-tuning and achieved surprising performance on multiple embodied visual navigation tasks.

There is also some work showing the use of a pretrained visual encoder can improve the performance of navigation agents in real environments. In [29], a pretrained DINO [30] was used as the visual encoder and fine-tuned on 70 RGB images with coarse semantic segmentation labels collected in a real environment. The results show that the robot was able to perform the visual navigation task well in a real environment. In [31], spatial autoencoders were used to pretrain the visual encoders on real environment images. The visual encoders with frozen weights were used in the learning of navigation policies during training in the simulated environment. The results show that the navigation policy had better sim2real performance on visual navigation.

### 2.3. Masked Autoencoders in Reinforcement Learning and Robotics

Masked Autoencoders (MAEs) [32] are self-supervised pretraining models based on an encoder–decoder structure that enable the encoder to learn visual representations by reconstructing the masked image. During pretraining, an MAE randomly masks the image patches with a fixed ratio, then the unmasked image patches are input to the encoder to extract latent representations, and finally, the decoder receives latent representations to reconstruct the original image. After the pretraining, the encoder will be used for downstream tasks. MAEs are now widely used in computer vision tasks [33] and have been shown to be a robust data augmentation method [34].

Although related work is relatively scarce, MAEs have also been used in reinforcement learning and robotics. In [35], an MAE was pretrained with real environment images, and the encoder with frozen weights was directly used as a visual encoder for a variety of downstream motor control tasks without any task-specific fine-tuning. The results show that the performance of the MAE pretrained encoder is significantly improved compared with the supervised encoder for downstream motor control tasks. In [36], an MAE with CNN-based models was compared on image-based reinforcement learning control tasks, and it was shown that the MAE was able to outperform CNN-based models on some tasks.

## 3. Method

### 3.1. Task Definition

**Figure 2:**
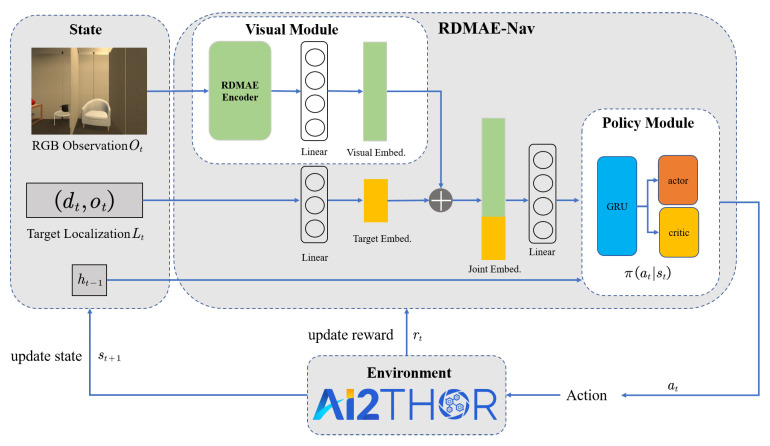
Overview architecture of RDMAE-Nav framework.

In the PointGoal navigation task, the agent must navigate to the target position by using RGB observations and GPS+Compass localization only. Concretely, the agent is initialized with a random location and orientation at the beginning of the episode. As shown in Figure 2, at each time step *t*, the agent obtains RGB observation $O_{t}$ from the monocular camera and target localization information $L_{t}$ from GPS+Compass, respectively. In particular, the localization information $L_{t}$ consists of two parts, $d_{t}$ and $o_{t}$, where $d_{t}$ is the distance of the target relative to the agent, and $o_{t}$ is the orientation of the target relative to the agent. Given the visual information $O_{t}$, the localization information $L_{t}$, and previous hidden information $h_{t-1}$, the agent is required to learn an optimal navigation policy $\pi \left(a_{t}|s_{t}\right)$ for the output action $a_{t}$ via DRL, where the state space is $s_{t}=\left\{O_{t},L_{t},h_{t-1}\right\}$, and the action space $a_{t}$ contains four discrete actions, namely $\left\{move\;\;forward\;\;0.25\;m,\;turn\;\;left\;\;30^{\circ },\;turn\;\;right\;\;30^{\circ },\;stop\right\}$. Overall, the episode is considered a success if the agent issues a stop action within 0.2 m of the target within a maximum of 300 steps. Otherwise, it is considered a failure. Note that the geodesic distance is used in the evaluation.

Noteworthily, previous works focus on the generalization to novel environments [8,9,10,11,12]. Nevertheless, the robustness of embodied navigation agents to visual corruptions also plays a crucial role in the navigation policy. ROBUSTNAV [13], as the first benchmark platform for robustness evaluation of embodied navigation agents, provides a number of solutions for visual corruptions. As one step further, we emphasize that the navigation policy should pay attention to generalization as well as robustness to various visual corruptions as the optimal strategy to follow. To this end, we propose the robust embodied navigation framework, RDMAE-Nav, with the details described in the following sections.

### 3.2. Overall Architecture of RDMAE-Nav

The overall architecture of the proposed RDMAE-Nav is shown in Figure 2. The framework follows the DRL pipeline, with the state space formed by the agent’s egocentric RGB observation, the target localization, and the previous hidden states. AI2THOR [2] is chosen as the simulation platform, which updates the state and outputs a reward for the next training step of DRL.

In the visual module, the visual encoder is the parameter-fixed encoder from a pretrained Regularized Denoising Mask AutoEncoder (RDMAE), which is depicted as RDMAE Encoder in Figure 2. The pretraining of the visual encoder is implemented by a self-supervised learning procedure with encoding–decoding as the auxiliary task. The pretrained visual encoder is involved in the succeeding navigation policy learning. A regularization term is imposed on the denoising paradigm by tackling both clean and noisy images from the RGB observations, which benefits the visual representation extraction for robust environment perception and understanding. The visual representations extracted by the visual encoder are projected as the visual embedding through a linear layer. Additionally, the target localization is projected as the target embedding by another linear layer. The visual embedding and the target embedding are aggregated as the joint embedding before being fed into the policy module.

The policy module includes a single-layer GRU and two single-layer MLPs, which are served as the actor and the critic of the DRL algorithm, respectively. The actor maps the output of GRU as the logits, which represent the discrete distribution of the actions, and the critic maps the output of GRU into a scalar value. The GRU network enables the memory ability of the policy network to consider historical experiences for future action determination. Decentralized Distributed Proximal Policy Optimization (DD-PPO) [8] is adopted as the DRL algorithm, which is a distributed Proximal Policy Optimization (PPO) [37] algorithm to accelerate the training process of the simulation environment effectively.

For DRL, the reward function $r_{t}$ is important to update the policy network, where $r_{t}=\left\{r_{success},r_{move}\right\}$. In our work, if the task is completed successfully at the time step *t*, the reward is set as 10, i.e., $r_{success}=10$; otherwise, the reward for moving one step $r_{move}$ is set as $\mathrm{GeoDist}\left(s_{t},a_{t}\right)-\mathrm{GeoDist}\left(s_{t-1},a_{t-1}\right)-0.01$, where −0.01 is the time penalty. Here, GeoDist denotes the geodesic distance from the agent’s current position to the target point. To sum up, the reward function $r_{t}$ can be written as

(1) $$r_{t}=\left\{\begin{array}{c} 10,\;\mathrm{if}\;\;\mathrm{success},\\ \mathrm{GeoDist}\left(s_{t-1},a_{t-1}\right)-\mathrm{GeoDist}\left(s_{t},a_{t}\right)-0.01,\;\mathrm{otherwise}. \end{array}\right.$$

### 3.3. Regularized Denoising Masked AutoEncoders (RDMAE) for Visual Pretraining

**Figure 3:**
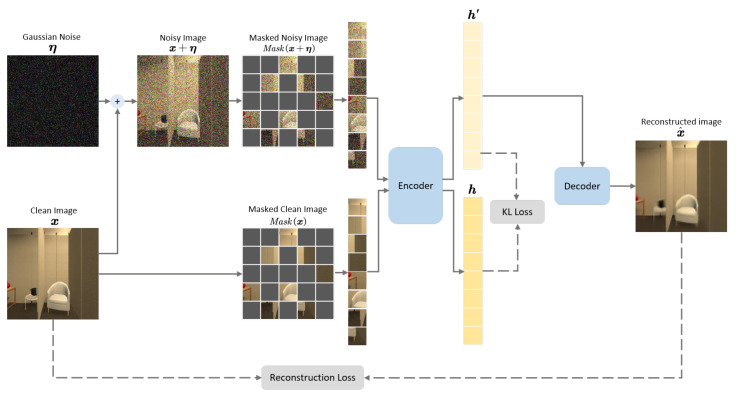
Illustration of RDMAE pretraining.

The visual encoder in RDMAE-Nav is pretrained by RDMAE, as shown in Figure 3. Assume x is the input clean image, and η is the Gaussian additive noise with noise level σ, where $\eta ∼N\left(0,\sigma^{2}I\right)$, I is the identity matrix. The Gaussian noise is added onto the clean image to generate a noisy image x+η. Both the noisy image x+η and the original clean one x are divided into the nonoverlapping patches, which are masked into Mask(x+η) and Mask(x), respectively, by a predefined masking ratio.

A self-supervised learning scheme is utilized to extract latent features for discriminative visual representations, with an auxiliary task constructed under the condition of insufficient labels. The autoencoder of the encoding–decoding mechanism is employed in the self-supervised learning scheme by following [15], with some modifications. Specifically, the encoder maps the input of both noisy and clean images into the low-dimensional feature space, and the decoder reconstructs the potential features of the noisy images back to the original clean images. Since both noisy and clean images are masked to be involved in the encode–decode process, a bidirectional Kullback–Leibler (KL) divergence loss is constructed to mitigate the representation distribution gap between them,

(2) $$L_{KL}=\frac{1}{2}\left(D_{KL}\left(h||h^{\prime }\right)+D_{KL}\left(h^{\prime }\left|\right|h\right)\right),$$

where h and $h^{\prime }$ denote the representation distributions of the masked clean images and noisy ones, respectively, and are defined as below:

(3) $$h=\mathrm{Encoder}\left(\mathrm{Mask}\left(x\right)\right),$$

(4) $$h^{\prime }=\mathrm{Encoder}\left(\mathrm{Mask}\left(x+\eta \right)\right).$$

$D_{KL}$ is the KL divergence to evaluate the difference between two distributions,

(5) $$D_{KL}\left(p\left(x\right)\left|\right|q\left(x\right)\right)=E_{x∼p\left(x\right)}\left[\log\frac{p\left(x\right)}{q\left(x\right)}\right],$$

where p(x) and q(x) stand for two probability distributions; E is the notation for expectation. The proposed bidirectional KL divergence loss is activated to mitigate the gap of representational distributions between clean and noisy images, which in turn is to achieve denoising implicitly.

The reconstruction procedure is also enabled in pretraining as the auxiliary denoising task by leveraging the reconstructed image $\hat{x}$. The reconstruction loss is implemented by calculating Mean Square Error (MSE) loss at the pixel level of all patches between the reconstructed image $\hat{x}$ and the clean image x by following [15],

(6) $$L_{recons}=\frac{1}{N}\sum_{i=1}^{N}{\left({\hat{x}}^{i}-x^{i}\right)}^{2},$$

where *N* is the number of pixels of each image.

The overall loss L is composed of the KL loss $L_{KL}$ and the reconstruction loss $L_{recons}$, which is expressed as

(7) $$L=L_{recons}+\alpha *L_{KL},$$

where α is the coefficient weight to control the KL loss.

Since image reconstruction and denoising are performed simultaneously, the visual encoder is capable of extracting discriminative semantic features as well as generalizing robustness against noises.

After pretraining, the decoder is discarded and the pretrained visual encoder is mounted in the RDMAE-Nav with the fixed parameters to make full use of its robustness in noises for the downstream navigation task with visual corruptions. Additionally, in the subsequent navigation policy learning, the masks are weeded out, and full sets of image patches are applied to the encoder.

### 3.4. Vision-Transformer-Based Visual Encoder

**Figure 4:**
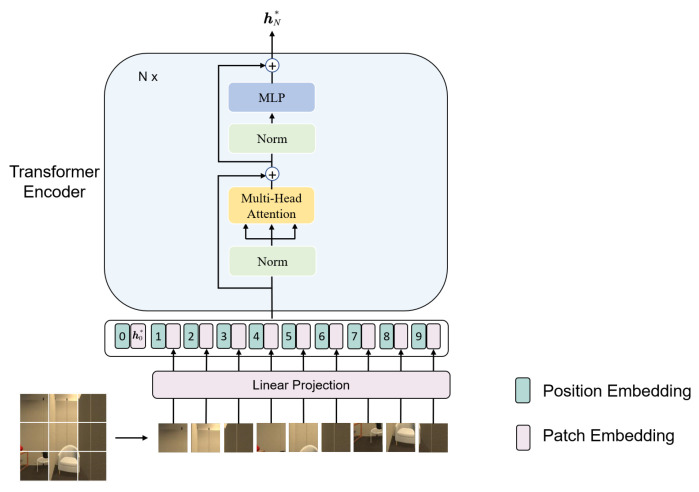
Network architecture of the ViT-based visual encoder.

The visual encoder of the proposed RDMAE leverages the powerful representation capability of the Vision Transformer [14], which was originally designed for image classification, for a more challenging visual navigation task. The difference between the visual encoder and the ViT is that only unmasked patches are applied to the visual encoder during pretraining. The visual encoder is based on the ViT, and they share the same network architecture, as detailed in Figure 4. The Transformer [38] is equipped with powerful visual representation capabilities endowed by a self-attention-based encoder–decoder. As mentioned previously, the input agent’s RGB observation is divided into fixed-size patches, each of which is projected as patch embedding by a linear layer. An extralearnable patch embedding is prepended to the sequence of patch embedding, marked as $h_{0}^{*}$ in Figure 4, whose final state $h_{N}^{*}$ through the Transformer encoder is adopted as the image representation for the downstream navigation task. Then, position embeddings are added to the patch embeddings and fed into the Transformer encoder. The Transformer encoder constitutes *N* stacked Transformer blocks, and each block consists of Multiheaded Attention (MHA) and MLP modules, where LayerNorm is applied before every module and residual connections are applied after every module. MHA allows the encoder to jointly attend to information of the input embeddings from different representation subspaces at different positions [38]. Therefore, the encoder is able to integrate information across the entire image and has stronger representation learning capabilities, which is beneficial to visual navigation.

## 4. Experiments

### 4.1. Simulation Platform

**Figure 5:**
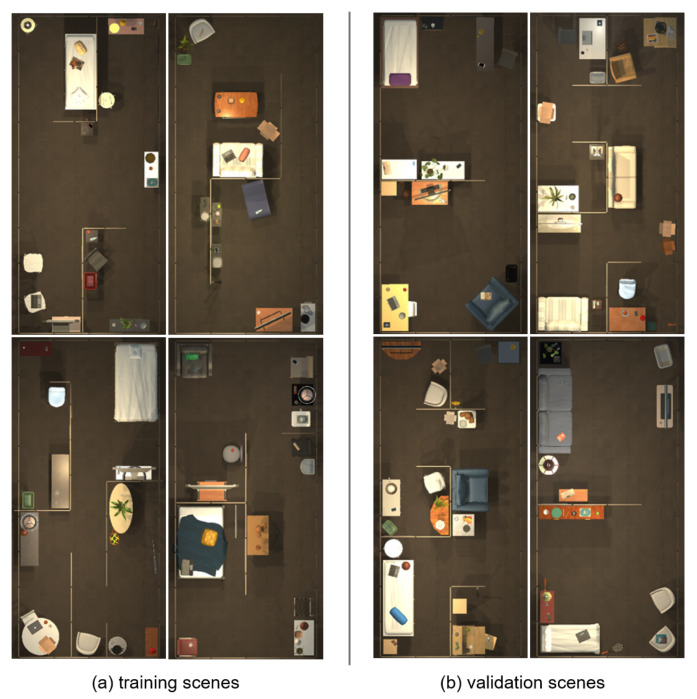
Top view of the selected scenes in the RoboTHOR dataset.

The experiments are conducted on the simulation platform AI2THOR [2] with the dataset RoboTHOR [39]. RoboTHOR contains 75 indoor scenes of 8.8 m × 3.9 m, of which 60 scenes are used for training (Figure 5a) and 15 scenes are used for validation (Figure 5b). There is a total of 108,000 different navigation tasks in the training scenes and 1100 different navigation tasks in the validation scenes. Some selected samples of scenes are shown in Figure 5. The validation scenes are set differently from the training scenes to evaluate the agent’s generalization to the novel scenes. The LoCoBot [40] robot is used as the navigation agent, which is equipped with an Intel RealSense camera.

### 4.2. Data Preraration

**Figure 6:**
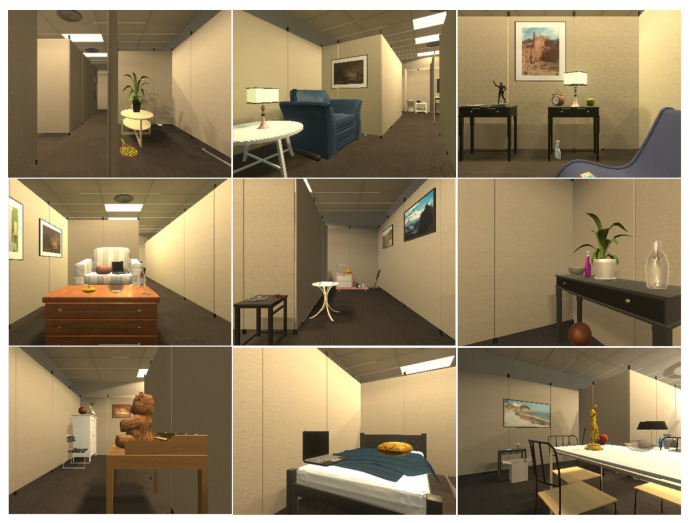
Agent egocentric RGB images for RDMAE visual pretraining.

For RDMAE visual pretraining, we collected 60k egocentric RGB images of the agent in a resolution of 300 × 400 from 60 training scenes of RoboTHOR, of which 1k images were collected for each scene. The data collection was accomplished by making the agent move randomly in the scene to save the egocentric images. Some of the collected images are shown in Figure 6.

### 4.3. Visual Corruptions Description

**Figure 7:**
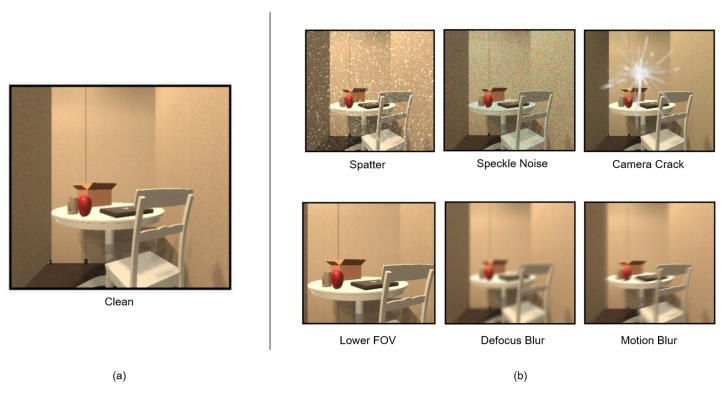
(**a**) The agent’s egocentric clean RGB observation. (**b**) The agent’s egocentric RGB observation in the presence of visual corruption.

In addition to evaluating the agent’s generalization ability to new scenes, we also evaluated its robustness to visual corruptions. The considered visual corruptions are Spatter, Speckle Noise, Camera Crack, Lower Fov, Motion Blur, and Defocus Blur, which are supported by ROBUSTNAV [13], as shown in Figure 7. Specifically, Spatter simulates the presence of camera lens occlusion, Speckle Noise simulates the inherent granular interference in the camera, Camera Crack simulates the presence of camera lens cracking, Lower Field Of View (FOV) requires the agent to use a lower camera FOV in evaluating (39.5°) than that used in training (79°), Motion Blur simulates blurred images caused by the agent jitters, Defocus Blur simulates the camera being out of focus, and Clean is the absence of visual corruption. By following ROBUSTNAV [13], the above visual corruptions are unseen by the agent during navigation policy training.

## 5. Experimental Results and Discussion

### 5.1. Evaluation Metrics

We adopt the commonly used Success Rate (SR) and Success weighted by Path Length (SPL) [5] as the evaluation metrics. During the evaluation, the agent is asked to navigate from the current position to a given target point, and SR indicates the success rate of navigation, which is

(8) $$\mathrm{SR}=\frac{1}{N}\sum_{i=1}^{N}S_{i},$$

where *N* denotes the number of evaluated tasks, and $S_{i}$ denotes whether the *i*-th task is successful, which is 1 if successful and 0 otherwise. SPL represents the ratio of the path length of the successful tasks to the shortest path length, which is

(9) $$\mathrm{SPL}=\frac{1}{N}\sum_{i=1}^{N}S_{i}\frac{l_{i}}{\max\left(p_{i},l_{i}\right)},$$

where $l_{i}$ is the shortest distance of the *i*-th task from the start to the target, and $p_{i}$ is the actual path length. A higher SR indicates a higher effectiveness of the navigation agent, and a higher SPL indicates higher efficiency.

In addition to the two common metrics mentioned above, we adopt another two metrics [13] for analyzing the agent’s behavior: average reward (R) and Dist2Target (Dist). R is the average reward obtained by the agent on evaluation, defined as below:

(10) $$R=\frac{1}{N}\sum_{i=1}^{N}r_{i},$$

where $r_{i}$ is the reward obtained by the agent of the *i*-th task. Dist is the average distance from the target when the agent issues the stop command, defined as below:

(11) $$\mathrm{Dist}=\frac{1}{N}\sum_{i=1}^{N}d_{i},$$

where $d_{i}$ is the distance from the target of the *i*-th task when the agent issues the stop command. In general, if the agent obtains a higher R and a lower Dist, the agent achieves more reasonable behavior.

### 5.2. RDMAE Pretraining Configuration

RDMAE is pretrained on the collected 60k agents’ egocentric images, all of which are resized to a fixed solution of 224 × 224. Unlike the original RDMAE, we use a smaller encoder–decoder structure based on ViT [14], which is ViT-S with an input patch size of 16 × 16. The ViT-S encoder consists of 12 Transformer blocks with embedding dimensions of 384, 6 attention heads, and an MLP multiplier of 4. ViT-S has about 22M parameters. The decoder uses 8 Transformer blocks with embedding dimensions of 256 and 16 attention heads. This asymmetric encoder–decoder structure ensures the encoder learns rich semantic features and reduces the pretraining time significantly [32].

This paper follows the mask ratio of 0.75, as in [32]. The learning rate is set as $1.5\times 10^{-4}$, the weight decay is 0.05, the batch size is set as 256, the epoch is 1000, and the noise level σ is set to 0.5. Empirically, the coefficient weight of KL Loss α is set as 0.1. The AdamW [41] optimizer with $\beta_{1}=0.9$ and $\beta_{2}=0.95$ is adopted. To prevent overfitting, we use simple data augmentation, including RandomResizedCrop with a crop ratio of (0.2, 1.0) and RandomHorizontalFlip. Two NVIDIA GeForce RTX 3090 GPUs are used for training.

**Figure 8:**
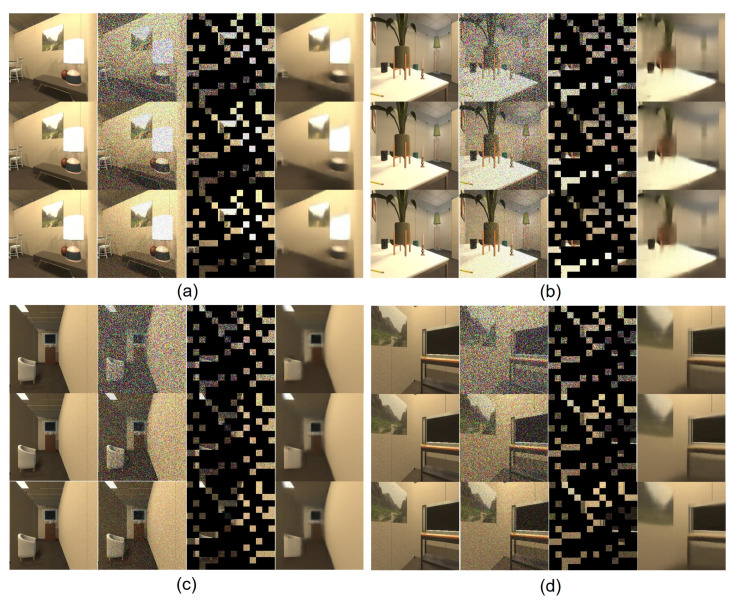
RDMAE reconstruction visualization. (**a**–**d**) are from four different scenes in the RoboTHOR validation set. For each image from left to right are: the original image, the Gaussian noise image (from top to bottom noise level σ = 0.5, 0.25, 0.1), the masked Gaussian noise image, and the reconstructed image.

After training, the RDMAE visualization of the reconstructed images is shown in Figure 8a–d from four different scenes in the RoboTHOR validation set. For each image from left to right are the original image, the Gaussian noise image (from top to bottom noise level σ = 0.5, 0.25, 0.1), the masked Gaussian noise image, and the reconstructed image. It is worth noting that the reconstructed images we show here are from the RoboTHOR validation set, which has not been seen by the agent during RDMAE training. Compared with the original clean image, RDMAE is able to reconstruct the masked noisy image well, indicating that RDMAE is able to extract robust features.

### 5.3. RDMAE-Nav Training Configuration

The procedure of RDMAE-Nav training is as follows. The agent’s egocentric RGB observation with a fixed resolution of 300 × 400 is resized to 224 × 224 before being fed into the visual module. The RGB observation is encoded into a 384 dim embedding by the pretrained visual encoder with frozen weights and then projected into a 512 dim visual embedding by a linear layer. The target localization is projected into a 32 dim target embedding by another linear layer. Next, the visual embedding and the target embedding are combined into a 544 dim joint embedding. The joint embedding is projected as a 1568-dimensional vector by a linear layer and subsequently fed into the GRU with 512 hidden units, along with the previous hidden state. The GRU outputs a 512 dim vector and the next hidden state. Finally, the 512 dim vector is received by two separate MLPs, which output 4 dim action logits and a scalar value, respectively.

Referring to [13], the learning rate is set as $3\times 10^{-4}$ and declines linearly. The discount factor is set to 0.99, and the GAE parameter is set to 0.95. The length of the rollout is 128. The optimizer uses Adam [42]. The DD-PPO clip parameter is set to 0.1. The epochs of DD-PPO is set to 4. For a fair comparison with [13], the navigation policy was trained for a total of 75M frames, in line with itself. Training was performed on a single NVIDIA GeForce RTX2060 GPU.

### 5.4. Performance on Visual Corruptions

We compare our method with the following approaches proposed in ROBUSTNAV.

(1) ROBUSTNAV is the standard approach to evaluate the benchmarks by using ResNet18 to extract visual features.
(2) ROBUSTNAV+AP is based on ROBUSTNAV by introducing an additional action prediction self-supervised task to resist visual corruptions.
(3) ROBUSTNAV+AP+SS-Adapt is based on ROBUSTNAV+AP by introducing self-supervised adaptation on specific corruptions (Spatter, Camera Crack, Lower-FOV, and Defocus Blur).
(4) ROBUSTNAV+RP is based on ROBUSTNAV by introducing an additional rotation prediction task to resist visual corruptions.
(5) ROBUSTNAV+RP+SS-Adapt is based on ROBUSTNAV+RP by introducing the self-supervised adaptation on specific corruptions (Spatter, Camera Crack, Lower-FOV, and Defocus Blur).
(6) ROBUSTNAV+Data Aug introduces various data augmentation methods during training. For more information about the above approaches, please refer to [13].

The comparisons on visual corruptions range from Clean, Spatter, Speckle Noise, Camera Crack, Lower Fov, Defocus Blur, and Motion Blur. Among the above corruptions, Spatter, Speckle Noise, Defocus Blur, and Motion Blur are classified into five severity levels, where level 5 indicates the most severe corruptions. For a fair comparison, the adjustable corruptions are set as level 5 by following ROBUSTNAV [13].

Table 1 shows the performance comparison of the navigation methods under clean and visual corruptions. For Spatter, compared with the suboptimal methods ROBUSTNAV+RP+SS-Adapt, which are 61.06% in SR and 47.16% in SPL, the proposed RDMAE-Nav improves the performance in both metrics by large margins, which are 89.26% in SR and 70.84% in SPL. The absolute improvement is 28.2% in SR and 23.68% in SPL. Similar results are observed in Speckle Noise and Defocus Blur. For Speckle Noise, compared with the suboptimal methods ROBUSTNAV+RP (under the SR metric) and ROBUSTNAV+Data Aug (under the SPL metric), the RDMAE-Nav improves the metric SR by 2.28% and the metric SPL by 6.41%. For Defocus Blur, compared with the suboptimal methods ROBUSTNAV+AP (under the SR metric) and ROBUSTNAV+RP+SS-Adapt (under the SPL metric), the RDMAE-Nav improves the metric SR by 9.46% and the metric SPL by 9.55%. For Cam Crack, Lower-FOV, and Motion Blur, the proposed RDMAE-Nav achieves comparable performance as well. Moreover, a remarkable observation is that for Defocus Blur and Motion Blur, mere performance degradation is observed compared with that in a clean environment. To sum up, the proposed RDMAE-Nav shows robustness against various visual corruptions.

To further analyze the agent behavior of the proposed RDMAE-Nav, we conducted the experiments under the metrics of R and Dist, and the results are shown in Table 2. Exemplarily, for Spatter, RDMAE-Nav exceeds the suboptimal method ROBUSTNAV+RP+SS-Adapt by 3.224 with 8.238 compared with 5.014, while several methods even pose a negative reward. In addition, RDMAE-Nav exhibits the lowest Dist of 0.5502. Similar conclusions can be drawn for Speckle Noise and Defocus Blur. For Cam Crack, Lower-FOV, and Motion Blur, RDMAE-Nav also achieves comparable performance. In the presence of visual corruption, RDMAE-Nav obtains higher rewards and stops closer to the target, which indicates that it makes more reasonable decisions.

**Figure 9:**
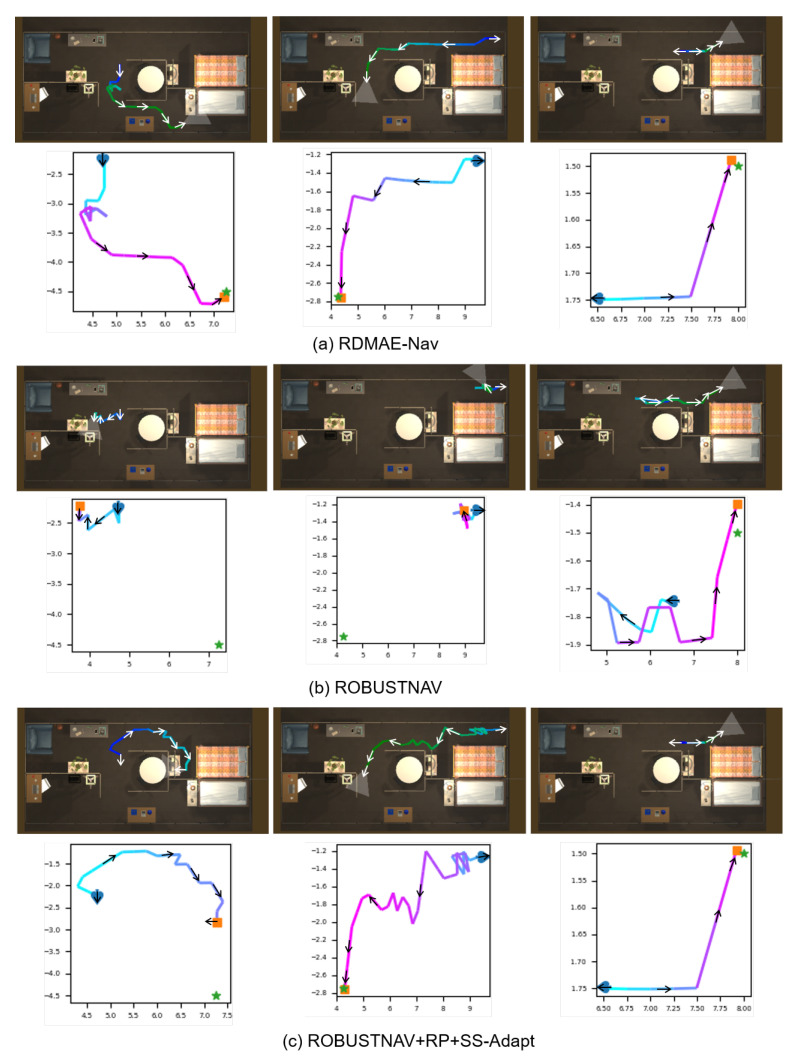
Examples of evaluated navigation trajectory visualization of RDMAE-Nav, ROBUSTNAV, and ROBUSTNAV+RP+SS-Adapt under Spatter.

Additionally, examples of the evaluated navigation trajectory visualization of RDMAE-Nav, ROBUSTNAV, and ROBUSTNAV+RP+SS-Adapt under Spatter are shown in Figure 9. The blue cardinal represents the agent’s initial position and orientation, the orange square represents the agent’s stop position, and the pentagram represents the target. Compared with ROBUSTNAV, RDMAE-Nav shows superior navigation performance in the presence of Spatter. Compared with ROBUSTNAV+RP+SS-Adapt, even though it reaches the target (middle part of Figure 9c), its actual path is longer compared with RDMAE-Nav (middle part of Figure 9a).

### 5.5. Ablations

#### 5.5.1. Contributions of KL Loss

**Figure 10:**
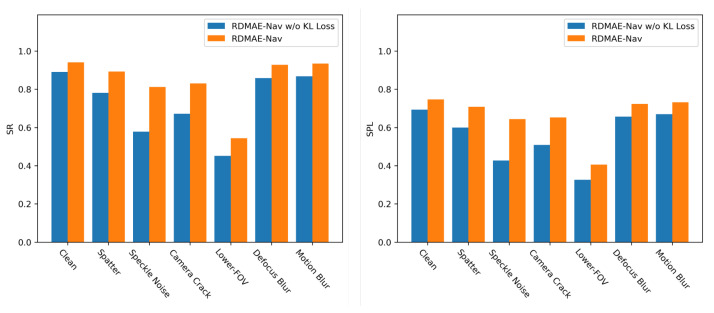
Histogram of contributions of KL Loss on the navigation performance of RDMAE-Nav.

The proposed RDMAE-Nav introduces a KL-divergence between the representation distribution of clean and noisy images. In this study, we specifically analyzed the contributions of KL Loss. This ablation removes the $L_{KL}$ loss of the visual encoder and only keeps the $L_{recons}$ loss for pretraining. The results are shown in Figure 10 and Table 3.

It is known from Figure 10 that introducing the KL Loss into the pretraining for the visual encoder improves the performance of RDMAE-Nav both in clean and various visual corruptions. In detail, for Speckle Noise, the performance improvement is the most significant by introducing KL Loss to RDMAE-Nav, with absolute improvements of 23.48% in SR and 21.63% in SPL, as in Table 3. For other corruptions, there are also varying degrees of performance improvement. From Table 4, RDMAE-Nav obtains higher R and lower Dist compared with RDMAE-Nav without KL Loss, which indicates that the introduction of KL Loss makes the navigation agent have more reliable and robust decisions in the presence of visual corruption.

#### 5.5.2. Contributions of Regularized Denoising

**Figure 11:**
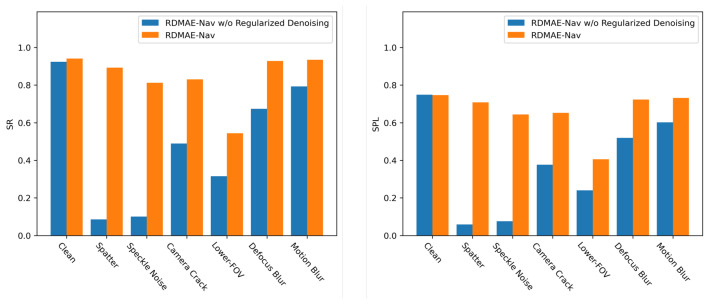
Histogram of contributions of Regularized Denoising on the navigation performance of RDMAE-Nav.

Note that the visual encoder of RDMAE-Nav is pretrained by a Regularized Denoising reconstruction task. In this study, we specifically analyzed the contributions of Regularized Denoising. This ablation removes Regularized Denoising during pretraining of the visual encoder. To conform with the configuration, we do not add Gaussian noise to the clean image and reconstruct the masked clean image accordingly by the encoder–decoder. The results are shown in Figure 11 and Table 5.

Basically, RDMAE-Nav without Regularized Denoising is on par with the complete version of RDMAE-Nav in a clean environment. However, in the presence of visual corruption, the performance of RDMAE-Nav without Regularized Denoising degrades dramatically. For Spatter, the SR and SPL of RDMAE-Nav without Regularized Denoising are both extremely low, only 8.553% and 5.923%, respectively, while the proposed Regularized Denoising mechanism makes great contributions to boosting performance. Similar results are observed in the other visual corruptions. From Table 6, RDMAE-Nav obtains higher R and lower Dist compared with RDMAE-Nav whiteout Regularized Denoising, which indicates that the introduction of Regularized Denoising makes the agent navigation have more reasonable decisions in the presence of visual corruption. It is worth noting that the Regularized Denoising mechanism has a significant improvement for Spatter and Speckle Noise, probably because these two corruptions are close to Gaussian noise, and RDMAE-Nav obtains stronger resistance after pretraining.

## 6. Conclusions

In this work, we proposed a robust PointGoal navigation framework, called RDMAE-Nav, which is robust to various visual corruptions. We designed a self-supervised pretraining method to enable the visual encoder of RDMAE-Nav to learn robust representations, which is called Regularized Denoising Masked AutoEncoders (RDMAE). Thanks to a denoising reconstruction task introduced in the RDMAE, RDMAE-Nav achieves robust representation ability. To the best of our knowledge, RDMAE-Nav is the first attempt to apply denoising masked visual pretraining in the PointGoal navigation task. Furthermore, RDMAE constructs a regularization loss by calculating the bidirectional Kullback–Leibler divergence between clean and noisy image representations, which mitigates the gap between them and thus enables the visual encoder of RDMAE-Nav to learn more robust representations. Extensive experiments on ROBUSTNAV, the benchmark platform for robustness evaluation for embodied visual navigation, demonstrate that RDMAE-Nav exhibits competitive robust performance compared with the state of the art.

Future works include the following aspects. Although RDMAE-Nav shows great advances in various visual corruptions, the performance still declines conspicuously compared with that in a clean environment. Therefore, more efforts are needed to alleviate the performance drop. Moreover, the structure of the visual encoder can be further improved to maximize the performance of RDMAE. Furthermore, evaluating the robustness of RDMAE-Nav in real-world scenarios is challenging but important work. Finally, the reason why Regularized Denoising masked visual pretraining shows different performance improvements for different visual corruptions deserves deeper research.

## Figures and Tables

**Table 1:** Navigation performance comparison under clean and visual corruption. The comparative performance metrics here are SR and SPL, with higher SR and SPL reflecting the higher effectiveness and efficiency of the navigation method.

Approach					Visual Corruption			
		Clean	Spatter	Speckle Noise	Camera Crack	Lower-FOV	Defocus Blur	Motion Blur
ROBUSTNAV	SR	98.82	33.58	67.42	82.07	42.49	75.89	95.72
	SPL	83.13	24.72	48.57	63.83	31.73	53.55	73.37
ROBUSTNAV+AP	SR	98.45	20.38	65.61	72.70	45.68	83.35	94.81
	SPL	83.28	15.70	47.03	56.82	35.14	61.51	74.3
ROBUSTNAV+AP+SS-Adapt	SR	37.31	14.19	\	57.87	32.94	40.95	\
	SPL	31.03	10.29	\	46.72	26.09	33.35	\
ROBUSTNAV+RP	SR	98.73	23.48	78.98	67.06	44.95	32.21	91.63
	SPL	82.53	18.63	55.92	53.70	32.74	22.47	65.27
ROBUSTNAV+RP+SS-Adapt	SR	94.63	61.06	\	60.42	50.59	79.16	\
	SPL	77.25	47.16	\	49.37	36.10	62.74	\
ROBUSTNAV+Data Aug	SR	98.45	23.93	77.25	88.44	71.70	81.26	96.91
	SPL	81.08	18.41	57.95	71.57	54.54	61.32	75.97
RDMAE-Nav	SR	94.09	89.26	81.26	83.08	54.41	92.81	93.45
	SPL	74.64	70.84	64.36	65.27	40.58	72.29	73.22

**Table 2:** Agent behavior analysis of the proposed RDMAE-Nav by comparing R and Dist metric. In general, if the agent obtains a higher R and a lower Dist, the agent achieves more reasonable behavior.

Approach					Visual Corruption			
		Clean	Spatter	Speckle Noise	Camera Crack	Lower-FOV	Defocus Blur	Motion Blur
ROBUSTNAV	R	9.513	0.458	3.577	5.043	2.321	5.425	8.017
	Dist	0.1393	3.306	2.2	1.554	2.115	1.468	0.6826
ROBUSTNAV+AP	R	9.51	−0.08434	5.217	6.047	3.053	7.349	8.937
	Dist	0.1316	3.673	1.652	1.289	2.115	0.8785	0.349
ROBSUTNAV+AP+SS-Adapt	R	1.478	−1.193	\	4.175	1.124	6.834	\
	Dist	2.591	3.397	\	1.971	3.082	1.126	\
ROBUSTNAV+RP	R	9.602	0.1546	6.816	5.29	2.843	1.165	8.411
	Dist	0.09848	4.052	1.164	1.65	2.31	2.961	0.4539
ROBUSTNAV+RP+SS-Adapt	R	9.046	5.014	\	4.506	3.524	6.741	\
	Dist	0.3248	1.838	\	1.88	1.965	1.136	\
ROBUSTNAV+Data Aug	R	9.465	0.08564	6.662	8.11	6.006	6.722	9.183
	Dist	0.1531	3.811	1.077	0.5868	1.245	1.015	0.1957
RDMAE-Nav	R	8.895	8.238	7.253	7.459	3.79	8.687	8.778
	Dist	0.3458	0.5502	0.9784	0.9005	1.982	0.3905	0.3561

**Table 3:** Detail of contributions of KL Loss on the navigation performance of RDMAE-Nav.

Approach					Visual Corruption			
		Clean	Spatter	Speckle Noise	Camera Crack	Lower-FOV	Defocus Blur	Motion Blur
RDMAE-Nav w/o KL Loss	SR	89.08	78.16	57.78	67.15	45.13	85.81	86.81
	SPL	69.36	60	42.73	50.89	32.62	65.65	66.91
**RDMAE-Nav**	SR	**94.09**	**89.26**	**81.26**	**83.08**	**54.41**	**92.81**	**93.45**
	SPL	**74.64**	**70.84**	**64.36**	**65.27**	**40.58**	**72.29**	**73.22**

**Table 4:** Contributions of KL Loss on the navigation behavior of RDMAE-Nav by comparing R and Dist metric.

Approach					Visual Corruption			
		Clean	Spatter	Speckle Noise	Camera Crack	Lower-FOV	Defocus Blur	Motion Blur
RDMAE-Nav w/o KL Loss	R	8.198	6.809	4.229	5.419	2.616	7.786	7.906
	Dist	0.5581	1.022	2.013	1.531	2.366	0.7601	0.7233
**RDMAE-Nav**	R	**8.895**	**8.238**	**7.253**	**7.459**	**3.79**	**8.687**	**8.778**
	Dist	**0.3458**	**0.5502**	**0.9784**	**0.9005**	**1.982**	**0.3905**	**0.3561**

**Table 5:** Detail of contributions of Regularized Denoising on navigation performance of RDMAE-Nav.

Approach					Visual Corruption			
		Clean	Spatter	Speckle Noise	Camera Crack	Lower-FOV	Defocus Blur	Motion Blur
RDMAE-Nav w/o Regularized Denoising	SR	92.36	8.553	10.1	48.95	31.57	67.42	79.34
	SPL	**74.94**	5.923	7.656	37.67	23.99	51.95	60.14
**RDMAE-Nav**	SR	**94.09**	**89.26**	**81.26**	**83.08**	**54.41**	**92.81**	**93.45**
	SPL	74.64	**70.84**	**64.36**	**65.27**	**40.58**	**72.29**	**73.22**

**Table 6:** Contributions of Regularized Denoising on the navigation behavior of RDMAE-Nav by comparing R and Dist metric.

Approach					Visual Corruption			
		Clean	Spatter	Speckle Noise	Camera Crack	Lower-FOV	Defocus Blur	Motion Blur
RDMAE-Nav w/o Regularized Denoising	R	8.678	−1.909	−1.715	3.166	0.9692	5.465	6.941
	Dist	0.3938	3.671	3.44	2.428	2.853	1.583	1.096
**RDMAE-Nav**	R	**8.895**	**8.238**	**7.253**	**7.459**	**3.79**	**8.687**	**8.778**
	Dist	**0.3458**	**0.5502**	**0.9784**	**0.9005**	**1.982**	**0.3905**	**0.3561**

## Data Availability

The data in this study are available from the first author upon request.
